# Crystal structure of 2-[(3*S*,4*S*)-4-(anthracen-9-yl)-1-(4-meth­oxy­phen­yl)-2-oxoazetidin-3-yl]-2-aza-2*H*-phenalene-1,3-dione unknown solvate

**DOI:** 10.1107/S2056989015002959

**Published:** 2015-02-13

**Authors:** Ísmail Çelik, Mehmet Akkurt, Aliasghar Jarrahpour, Javad Ameri Rad, Ömer Çelik

**Affiliations:** a Department of Physics, Faculty of Arts and Sciences, Cumhuriyet University, 06532 Sivas, Turkey; b Department of Physics, Faculty of Sciences, Erciyes University, 38039 Kayseri, Turkey; c Department of Chemistry, College of Sciences, Shiraz University, 71454 Shiraz, Iran; d Department of Physics, Faculty of Education, Dicle University, 21280, Diyarbakir, Turkey; e Science and Technology Application and Research Center, Dicle University, 21280, Diyarbakir, Turkey

**Keywords:** crystal structure, β-lactam ring, 2-azetidinone, anthracene, intra­molecular C—H⋯N hydrogen bond, C—H⋯π inter­actions, π–π stacking inter­actions

## Abstract

The central β-lactam ring of the title compound, C_36_H_24_N_2_O_4_, is almost planar (r.m.s. deviation = 0.003 Å) and makes dihedral angles of 17.17 (19), 89.76 (17) and 78.44 (17)° with the benzene ring, the anthracene ring (r.m.s. deviation = 0.003 Å) and the 1*H*-benzo[*de*]iso­quinoline-1,3(2*H*)-dione moiety, which is nearly planar [maximum deviation = 0.098 (2) Å], respectively. The mol­ecular structure is stabilized by an intra­molecular C—H⋯N hydrogen bond. In the crystal, mol­ecules are linked *via* C—H⋯π and π–π stacking inter­actions [centroid–centroid distances = 3.5270 (19) and 3.779 (2) Å], forming a three-dimensional structure. A region of disordered electron density, probably disordered solvent mol­ecules, was treated with the SQUEEZE procedure in *PLATON* [Spek (2015 ▸). *Acta Cryst.* C**71**, 9–18], which indicated a solvent cavity of 322 Å^3^ containing approximately 91 electrons. Their formula mass and unit-cell characteristics were not taken into account during the refinement.

## Related literature   

For general background to β-lactams and their biological properties, see: Fischbach & Walsh (2009 ▸); Georg (1992 ▸); Kim *et al.* (2014 ▸); Ocampo & Dolbier (2004 ▸); Palomo *et al.* (2004 ▸); Smith *et al.* (2014 ▸); Soengas *et al.* (2011 ▸); von Nussbaum *et al.* (2006 ▸); Walsh & Wencewicz (2014 ▸). For related structures, see: Çelik *et al.* (2015 ▸); Atioğlu *et al.* (2014 ▸); Butcher *et al.* (2011 ▸); Jarrahpour *et al.* (2012 ▸); Jarrahpoor & Khalili (2007 ▸); Jarrahpour & Ebrahimi (2010 ▸). For details of the SQUEEZE procedure in *PLATON*, see: Spek (2015 ▸).
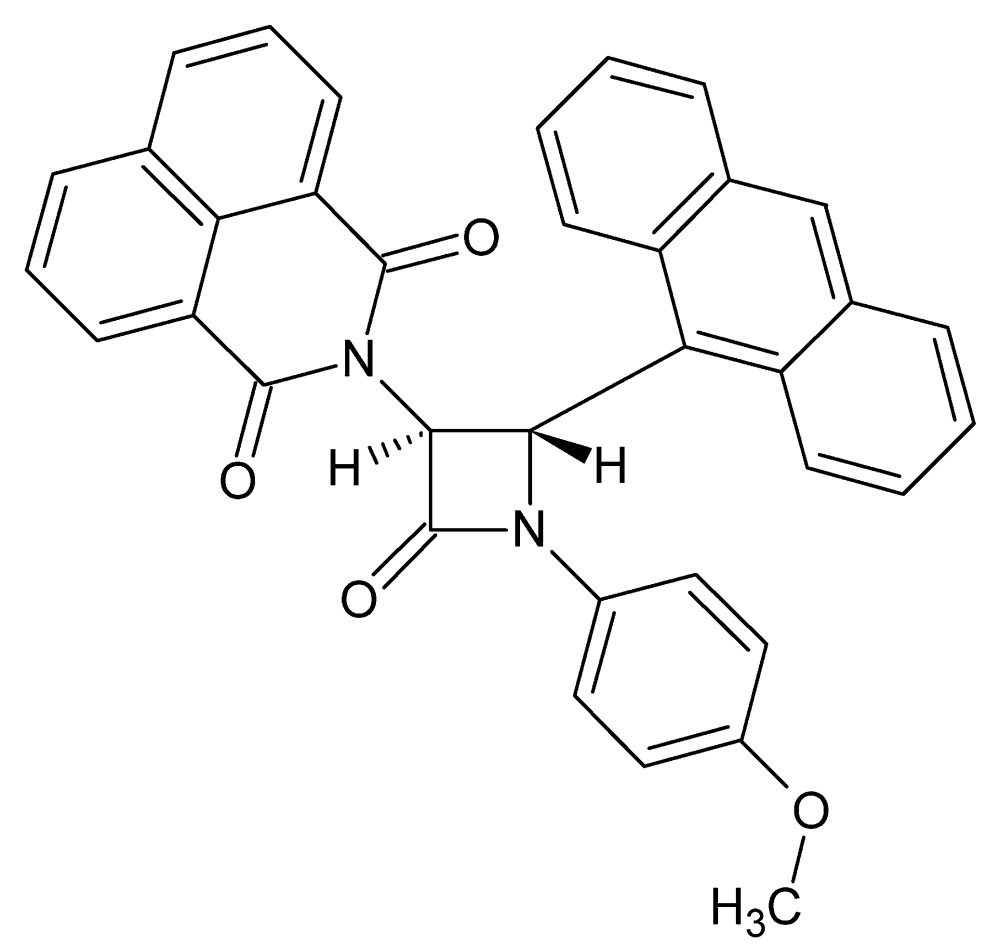


## Experimental   

### Crystal data  

C_36_H_24_N_2_O_4_
*M* *_r_* = 548.57
Monoclinic, 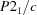

*a* = 9.9880 (1) Å
*b* = 29.1281 (4) Å
*c* = 11.0751 (2) Å
β = 101.367 (1)°
*V* = 3158.89 (8) Å^3^
*Z* = 4
Mo *K*α radiation
μ = 0.08 mm^−1^
*T* = 296 K
0.35 × 0.20 × 0.15 mm

### Data collection  

Bruker APEXII CCD diffractometer
33697 measured reflections
6431 independent reflections
3502 reflections with *I* > 2σ(*I*)
*R*_int_ = 0.045

### Refinement  

*R*[*F* ^2^ > 2σ(*F* ^2^)] = 0.072
*wR*(*F* ^2^) = 0.242
*S* = 1.10
6431 reflections
349 parameters
2 restraints
H-atom parameters constrained
Δρ_max_ = 0.39 e Å^−3^
Δρ_min_ = −0.30 e Å^−3^

### 

Data collection: *APEX2* (Bruker, 2007 ▸); cell refinement: *SAINT* (Bruker, 2007 ▸); data reduction: *SAINT*; program(s) used to solve structure: *SHELXS2014* (Sheldrick, 2008 ▸); program(s) used to refine structure: *SHELXL2014* (Sheldrick, 2015 ▸); molecular graphics: *ORTEP-3 for Windows* (Farrugia, 2012 ▸); software used to prepare material for publication: *SHELXL2014* and *PLATON* (Spek, 2009 ▸).

## Supplementary Material

Crystal structure: contains datablock(s) global, I. DOI: 10.1107/S2056989015002959/su5084sup1.cif

Structure factors: contains datablock(s) I. DOI: 10.1107/S2056989015002959/su5084Isup2.hkl

Supporting information file. DOI: 10.1107/S2056989015002959/su5084Isup3.cml

**Fig. 1.**
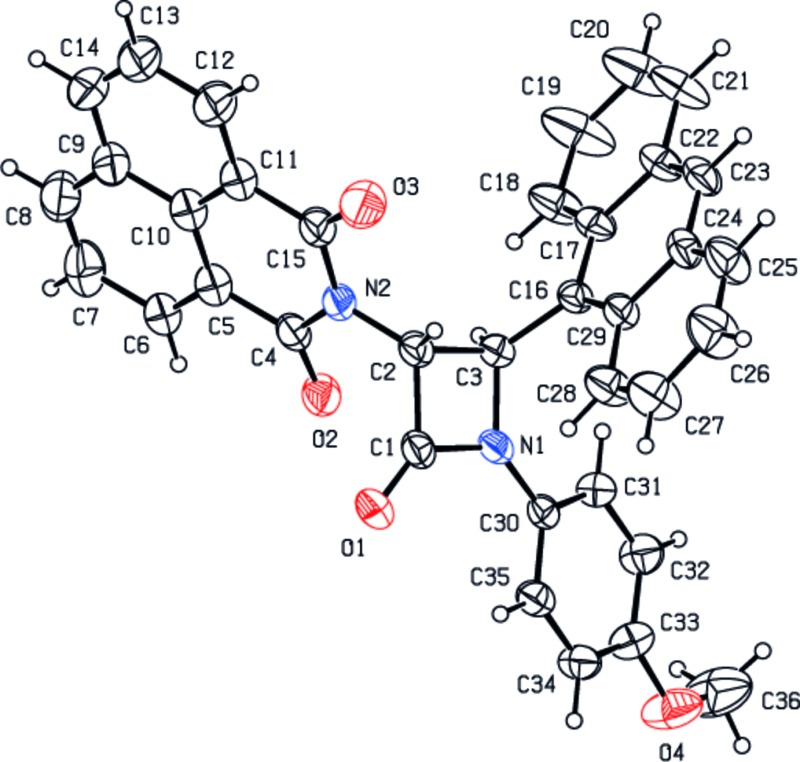
Perspective view of the molecular structure of the title compound, with atom labelling. Displacement ellipsoids are drawn at the 30% probability level.

**Fig. 2.**
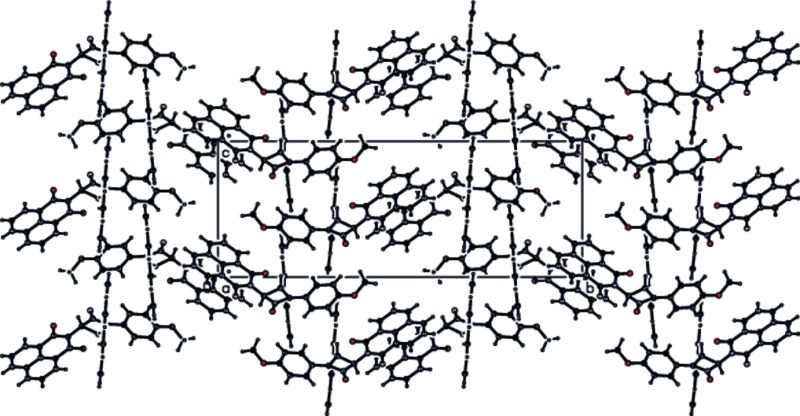
View along a-axis of the crystal packing of the title compound.

. DOI: 10.1107/S2056989015002959/su5084fig1.tif
Perspective view of the mol­ecular structure of the title compound, with atom labelling. Displacement ellipsoids are drawn at the 30% probability level.

a . DOI: 10.1107/S2056989015002959/su5084fig2.tif
View along *a*-axis of the crystal packing of the title compound.

CCDC reference: 1048898

Additional supporting information:  crystallographic information; 3D view; checkCIF report

## Figures and Tables

**Table 1:** Hydrogen-bond geometry (, ) *Cg*5 is the centroid of the C16/C17/C22C24/C29 benzene ring.

*D*H*A*	*D*H	H*A*	*D* *A*	*D*H*A*
C28H28N1	0.93	2.35	3.022(4)	129
C13H13*Cg*5^i^	0.93	2.84	3.713(4)	158

Symmetry code: (i) 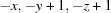.

## supplementary crystallographic information

### S1. Comment

β-Lactams are a very important class of heterocyclic compounds due to their obvious biological activity (Von Nussbaum, *et al.*, 2006). Because the β-lactam ring forms the main part of the penicillin structure, the β-lactam antibiotics have been the subject of much discussion and investigation (Soengas, *et al.*, 2011). However, due to their extensive use common infections are once again becoming deadly (Fischbach & Walsh, 2009) and there is still a significant need for new research on β-lactams (Walsh & Wencewicz, 2014). Furthermore, β-lactams have been used in the synthesis of a wide variety of compounds which contain a nitrogen atom in their structures (Kim *et al.*, 2014). In the past decades, a growing interest has been shown in the development of the various methods for the synthesis of β-lactams (Georg, 1992; Palomo *et al.*, 2004; Ocampo & Dolbier, 2004). It has been observed that the most direct synthetic way to access β-lactam rings remains the formal [2 + 2] cycloaddition of ketenes and imines (Staudinger reaction) [Smith *et al.*, 2014].

The β-lactam ring (N1/C1–C3) of the title compound, Fig. 1, is nearly planar [maximum deviation for atom N1 = 0.051 (2) Å]. It makes dihedral angles of 17.17 (19), 89.76 (17) and 78.44 (17)° with the benzene ring (C30–C35), the anthracene ring system (C16–C29) and the 1*H*-benzo[de]isoquinoline-1,3(2*H*)-dione (N2/O2/O3/C4–C15) moiety, respectively. The maximum deviations in the latter two ring systems are 0.050 (2) and 0.098 (2) Å, respectively.

All bond lengths and bond angles are normal and comparable with those reported for related compounds (Çelik *et al.*, 2015; Butcher *et al.*, 2011; Atioğlu *et al.*, 2014; Jarrahpour *et al.*, 2012; Jarrahpoor & Khalili, 2007; Jarrahpour & Ebrahimi, 2010). An intramolecular C—H···N hydrogen bond (Table 1) stabilizes the molecular conformation.

In the crystal, molecules are connected by C—H···π and π-π stacking interactions forming a three-dimensional structure; see Table 1 and Fig. 2 [*Cg*2···*Cg*3^i^ = 3.5270 (19) and *Cg*6···*Cg*7^ii^ = 3.779 (2) Å; where *Cg*2, *Cg*3, *Cg*6 and *Cg*7 are centroids of the N2/C4/C5/C10/C11/C15, C5–C10, C17–C22 and C24–C29 rings, respectively; symmetry codes: (i) -x+1, -y+1, -z+1; (ii) x, -y +1/2, z+1/2].

### S2. Experimental

1-(anthracen-9-yl)-*N*-(4-methoxyphenyl) methanimine (1 mmol), triethylamine (5 mmol), 2-(1,3-dioxo-1*H*-benzo[de]isoquinolin-2(3*H*)-yl) acetic acid (1.50 mmol) and tosyl chloride (1.50 mmol) were added to anhydrous CH_2_Cl_2_ (5 ml) and the mixture was stirred at room temperature for 24 h. The mixture was then washed with HCl 1N (2 × 20 ml), saturated aqueous NaHCO_3_ solution (50 ml) and brine (20 ml). The organic layer was dried (Na_2_SO_4_) and the solvent was removed in vacuo to give the product as a yellow solid. It was then purified by recrystallization from DMSO to afford yellow crystals (yield: 80%; m.p.: 458–460 K; IR (KBr, cm^-1^): 1751 (CO β-lactam),1704 (CO Naph), 1666 (CO Naph); ^1^H-NMR (250 MHz, DMSO-d_6_) δ: 3.57 (CH_3_ s, 3H), 6.73–6.79 (m, 4H), 6.97 (d, 2H, J = 2.5), 7.09 (aromatic d, 2H, J = 8.75), 7.49–7.57 (aromatic, m, 4H), 7.81–7.90 (aromatic, m, 2H), 8.16 (aromatic d, 2H, J = 7.75), 8.42–8.51 (aromatic, m, 6H), 8.51(aromatic s, 1H); ^13^C-NMR (62 MHz, DMSO-d_6_) δ 163.81 (CO β-lactam), 162.18 (CO Naph), 155.77, 134.93, 131.30, 131.18, 131.07, 130.96, 130.01, 129.84, 129.78, 127.52, 127.44, 127.25, 125.17, 124.14, 122.57, 121.41, 117.75, 114.55(aromatic carbons), 62.10 (C β-lactam), 55.58 (C β-lactam), 55.05 (CH_3_—O); GC—MS m/*z* = 548 [*M*+]; Analysis calculated for C_36_H_24_N_2_O_4_: C, 78.82; H, 4.41; N, 5.11%. Found: C, 76.53; H, 4.45; N, 5.81%.

### S3. Refinement

H atoms attached to C atoms were positioned geometrically (C—H = 0.93 - 0.98 Å), and refined using a riding model with *U*_iso_(H) = 1.5*U*_eq_(C) for methyl H atoms and = 1.2*U*_eq_(C) fro other H atoms. The crystal was of poor quality and did not diffract significantly at high 2θ angles, probably due to the presence of the disordered solvent molecules. 23 reflections were omitted owing to bad disagreement. A region of disordered electron density, most probably disordered solvent molecules, occupying voids of *ca* 322 Å^3^ for an electron count of 91, was removed with the SQUEEZE procedure in *PLATON* [Spek (2015). Acta Cryst. C71, 9-18] following unsuccessful attempts to model it as plausible solvent molecules. Their formula mass and unit-cell characteristics were not taken into account during refinement.

### Crystal data

**Table:**

C_36_H_24_N_2_O_4_	*F*(000) = 1144
*M**_r_* = 548.57	*D*_x_ = 1.153 Mg m^−^^3^
Monoclinic, *P*2_1_/*c*	Mo *K*α radiation, λ = 0.71073 Å
Hall symbol: -P 2ybc	Cell parameters from 4703 reflections
*a* = 9.9880 (1) Å	θ = 2.5–21.8°
*b* = 29.1281 (4) Å	µ = 0.08 mm^−^^1^
*c* = 11.0751 (2) Å	*T* = 296 K
β = 101.367 (1)°	Prism, yellow
*V* = 3158.89 (8) Å^3^	0.35 × 0.20 × 0.15 mm
*Z* = 4	

### Data collection

**Table:**

Bruker APEXII CCD diffractometer	3502 reflections with *I* > 2σ(*I*)
Radiation source: sealed tube	*R*_int_ = 0.045
Graphite monochromator	θ_max_ = 26.4°, θ_min_ = 1.4°
φ and ω scans	*h* = −12→12
33697 measured reflections	*k* = −36→36
6431 independent reflections	*l* = −13→13

### Refinement

**Table:**

Refinement on *F*^2^	2 restraints
Least-squares matrix: full	Hydrogen site location: inferred from neighbouring sites
*R*[*F*^2^ > 2σ(*F*^2^)] = 0.072	H-atom parameters constrained
*wR*(*F*^2^) = 0.242	*w* = 1/[σ^2^(*F*_o_^2^) + (0.1309*P*)^2^] where *P* = (*F*_o_^2^ + 2*F*_c_^2^)/3
*S* = 1.10	(Δ/σ)_max_ < 0.001
6431 reflections	Δρ_max_ = 0.39 e Å^−^^3^
349 parameters	Δρ_min_ = −0.30 e Å^−^^3^

### Special details

**Table:**

Geometry. Bond distances, angles *etc*. have been calculated using the rounded fractional coordinates. All su's are estimated from the variances of the (full) variance-covariance matrix. The cell e.s.d.'s are taken into account in the estimation of distances, angles and torsion angles
Refinement. Refinement on *F*^2^ for ALL reflections except those flagged by the user for potential systematic errors. Weighted *R*-factors *wR* and all goodnesses of fit *S* are based on *F*^2^, conventional *R*-factors *R* are based on *F*, with *F* set to zero for negative *F*^2^. The observed criterion of *F*^2^ > σ(*F*^2^) is used only for calculating -*R*-factor-obs *etc*. and is not relevant to the choice of reflections for refinement. *R*-factors based on *F*^2^ are statistically about twice as large as those based on *F*, and *R*-factors based on ALL data will be even larger.

### Fractional atomic coordinates and isotropic or equivalent isotropic displacement parameters (Å^2^)

**Table:**

	*x*	*y*	*z*	*U*_iso_*/*U*_eq_	
O1	0.4190 (2)	0.35039 (7)	0.24633 (19)	0.0692 (8)	
O2	0.46467 (19)	0.36965 (7)	0.52700 (19)	0.0693 (8)	
O3	0.0898 (2)	0.45145 (8)	0.3595 (3)	0.1045 (10)	
O4	0.4750 (3)	0.12563 (9)	0.3656 (3)	0.1069 (11)	
N1	0.2807 (2)	0.30426 (7)	0.3470 (2)	0.0543 (8)	
N2	0.2815 (2)	0.41138 (7)	0.4404 (2)	0.0572 (8)	
C1	0.3327 (3)	0.34368 (9)	0.3064 (3)	0.0563 (9)	
C2	0.2321 (3)	0.37334 (9)	0.3612 (3)	0.0554 (9)	
C3	0.1920 (2)	0.32787 (9)	0.4197 (3)	0.0526 (9)	
C4	0.4052 (3)	0.40621 (10)	0.5227 (3)	0.0588 (10)	
C5	0.4560 (3)	0.44602 (11)	0.5995 (3)	0.0728 (6)	
C6	0.5807 (3)	0.44420 (11)	0.6764 (3)	0.0728 (6)	
C7	0.6296 (4)	0.48285 (15)	0.7508 (4)	0.0961 (17)	
C8	0.5545 (5)	0.52161 (15)	0.7453 (4)	0.0965 (17)	
C9	0.4259 (4)	0.52484 (12)	0.6675 (3)	0.0839 (14)	
C10	0.3769 (3)	0.48620 (11)	0.5934 (3)	0.0728 (6)	
C11	0.2496 (3)	0.48927 (11)	0.5132 (3)	0.0728 (6)	
C12	0.1705 (4)	0.52855 (12)	0.5103 (5)	0.1133 (13)	
C13	0.2184 (4)	0.56608 (13)	0.5837 (5)	0.1133 (13)	
C14	0.3441 (5)	0.56448 (13)	0.6599 (4)	0.1056 (18)	
C15	0.1979 (3)	0.45068 (10)	0.4311 (3)	0.0724 (13)	
C16	0.0415 (2)	0.31708 (8)	0.4028 (2)	0.0495 (9)	
C17	−0.0176 (3)	0.31832 (10)	0.5074 (3)	0.0564 (9)	
C18	0.0568 (3)	0.32229 (14)	0.6311 (3)	0.0874 (13)	
C19	−0.0069 (4)	0.3238 (2)	0.7281 (4)	0.1318 (16)	
C20	−0.1489 (4)	0.3223 (2)	0.7123 (4)	0.1318 (16)	
C21	−0.2248 (3)	0.31779 (16)	0.5989 (4)	0.0983 (18)	
C22	−0.1647 (3)	0.31528 (10)	0.4932 (3)	0.0639 (11)	
C23	−0.2446 (3)	0.31168 (10)	0.3770 (3)	0.0657 (11)	
C24	−0.1887 (3)	0.30945 (10)	0.2726 (3)	0.0591 (10)	
C25	−0.2736 (3)	0.30603 (14)	0.1548 (3)	0.0866 (13)	
C26	−0.2207 (4)	0.30221 (16)	0.0529 (4)	0.1054 (18)	
C27	−0.0786 (4)	0.30242 (15)	0.0617 (3)	0.0986 (16)	
C28	0.0072 (3)	0.30741 (13)	0.1722 (3)	0.0786 (13)	
C29	−0.0427 (3)	0.31138 (9)	0.2845 (3)	0.0562 (10)	
C30	0.3253 (2)	0.25829 (9)	0.3516 (2)	0.0479 (8)	
C31	0.2942 (3)	0.22912 (10)	0.4384 (3)	0.0611 (10)	
C32	0.3417 (3)	0.18386 (11)	0.4453 (3)	0.0708 (11)	
C33	0.4222 (3)	0.16891 (11)	0.3658 (3)	0.0694 (11)	
C34	0.4512 (3)	0.19801 (11)	0.2776 (3)	0.0669 (11)	
C35	0.4034 (3)	0.24258 (10)	0.2700 (3)	0.0589 (10)	
C36	0.4632 (7)	0.09623 (15)	0.4623 (5)	0.140 (3)	
H2	0.15700	0.38340	0.29570	0.0660*	
H3	0.23420	0.32690	0.50740	0.0630*	
H6	0.63360	0.41780	0.68020	0.0870*	
H7	0.71420	0.48140	0.80390	0.1150*	
H8	0.58870	0.54660	0.79400	0.1160*	
H12	0.08480	0.52980	0.45890	0.1360*	
H13	0.16490	0.59230	0.58100	0.1360*	
H14	0.37590	0.59000	0.70750	0.1270*	
H18	0.15160	0.32390	0.64540	0.1050*	
H19	0.04530	0.32580	0.80730	0.1580*	
H20	−0.19060	0.32450	0.78010	0.1580*	
H21	−0.31930	0.31630	0.58930	0.1180*	
H23	−0.33910	0.31070	0.36870	0.0790*	
H25	−0.36790	0.30640	0.14770	0.1040*	
H26	−0.27820	0.29940	−0.02370	0.1270*	
H27	−0.04280	0.29910	−0.00920	0.1180*	
H28	0.10090	0.30830	0.17520	0.0940*	
H31	0.24110	0.23940	0.49320	0.0730*	
H32	0.31880	0.16390	0.50360	0.0850*	
H34	0.50360	0.18770	0.22240	0.0800*	
H35	0.42380	0.26210	0.20970	0.0710*	
H36A	0.50430	0.06720	0.45060	0.2110*	
H36B	0.50860	0.10950	0.53880	0.2110*	
H36C	0.36840	0.09170	0.46410	0.2110*	

### Atomic displacement parameters (Å^2^)

**Table:**

	*U*^11^	*U*^22^	*U*^33^	*U*^12^	*U*^13^	*U*^23^
O1	0.0567 (11)	0.0759 (14)	0.0813 (14)	−0.0105 (10)	0.0287 (11)	0.0031 (11)
O2	0.0529 (11)	0.0635 (13)	0.0879 (15)	0.0010 (9)	0.0048 (10)	0.0021 (10)
O3	0.0591 (14)	0.0813 (16)	0.162 (2)	0.0163 (12)	−0.0051 (16)	−0.0168 (16)
O4	0.146 (2)	0.0762 (16)	0.1096 (19)	0.0450 (15)	0.0521 (18)	0.0099 (15)
N1	0.0392 (11)	0.0551 (14)	0.0709 (15)	0.0010 (9)	0.0166 (11)	0.0024 (11)
N2	0.0437 (12)	0.0479 (13)	0.0800 (16)	−0.0018 (9)	0.0120 (11)	−0.0019 (11)
C1	0.0409 (14)	0.0601 (17)	0.0673 (18)	−0.0064 (12)	0.0093 (13)	0.0001 (14)
C2	0.0407 (13)	0.0516 (15)	0.0742 (18)	0.0021 (11)	0.0121 (13)	0.0030 (13)
C3	0.0440 (14)	0.0528 (15)	0.0634 (17)	0.0015 (11)	0.0162 (13)	−0.0020 (12)
C4	0.0447 (15)	0.0578 (18)	0.0767 (19)	−0.0046 (13)	0.0185 (14)	0.0030 (14)
C5	0.0643 (9)	0.0668 (10)	0.0908 (12)	−0.0112 (8)	0.0237 (8)	−0.0039 (9)
C6	0.0643 (9)	0.0668 (10)	0.0908 (12)	−0.0112 (8)	0.0237 (8)	−0.0039 (9)
C7	0.092 (3)	0.105 (3)	0.086 (3)	−0.034 (2)	0.005 (2)	−0.002 (2)
C8	0.109 (3)	0.090 (3)	0.096 (3)	−0.035 (2)	0.034 (3)	−0.020 (2)
C9	0.083 (2)	0.078 (2)	0.099 (3)	−0.0250 (19)	0.038 (2)	−0.0199 (19)
C10	0.0643 (9)	0.0668 (10)	0.0908 (12)	−0.0112 (8)	0.0237 (8)	−0.0039 (9)
C11	0.0643 (9)	0.0668 (10)	0.0908 (12)	−0.0112 (8)	0.0237 (8)	−0.0039 (9)
C12	0.0867 (19)	0.0623 (16)	0.197 (3)	0.0013 (14)	0.043 (2)	−0.0180 (18)
C13	0.0867 (19)	0.0623 (16)	0.197 (3)	0.0013 (14)	0.043 (2)	−0.0180 (18)
C14	0.114 (3)	0.066 (2)	0.153 (4)	−0.019 (2)	0.066 (3)	−0.032 (2)
C15	0.0497 (17)	0.0564 (18)	0.112 (3)	−0.0007 (13)	0.0181 (18)	−0.0051 (17)
C16	0.0392 (13)	0.0479 (14)	0.0624 (17)	0.0054 (10)	0.0124 (13)	0.0035 (12)
C17	0.0415 (14)	0.0648 (17)	0.0644 (18)	0.0123 (12)	0.0138 (13)	0.0084 (13)
C18	0.0500 (17)	0.143 (3)	0.070 (2)	0.0187 (19)	0.0141 (17)	0.006 (2)
C19	0.0717 (16)	0.255 (4)	0.0734 (18)	0.042 (2)	0.0260 (16)	0.025 (2)
C20	0.0717 (16)	0.255 (4)	0.0734 (18)	0.042 (2)	0.0260 (16)	0.025 (2)
C21	0.0488 (18)	0.162 (4)	0.092 (3)	0.023 (2)	0.0334 (19)	0.029 (2)
C22	0.0419 (15)	0.080 (2)	0.073 (2)	0.0101 (13)	0.0193 (15)	0.0148 (15)
C23	0.0366 (14)	0.077 (2)	0.085 (2)	0.0002 (13)	0.0157 (15)	0.0066 (16)
C24	0.0407 (14)	0.0654 (17)	0.0695 (19)	−0.0064 (12)	0.0071 (14)	0.0018 (14)
C25	0.0479 (17)	0.125 (3)	0.084 (2)	−0.0092 (18)	0.0056 (18)	−0.001 (2)
C26	0.069 (2)	0.163 (4)	0.076 (3)	−0.009 (2)	−0.006 (2)	−0.007 (2)
C27	0.074 (2)	0.156 (4)	0.064 (2)	−0.003 (2)	0.0090 (19)	−0.013 (2)
C28	0.0540 (18)	0.110 (3)	0.073 (2)	0.0051 (17)	0.0155 (17)	−0.0026 (18)
C29	0.0432 (14)	0.0580 (16)	0.0682 (19)	−0.0004 (12)	0.0133 (14)	0.0002 (13)
C30	0.0343 (12)	0.0510 (15)	0.0572 (15)	−0.0027 (10)	0.0062 (11)	−0.0019 (12)
C31	0.0573 (16)	0.0622 (18)	0.0669 (18)	0.0035 (13)	0.0201 (14)	−0.0074 (14)
C32	0.083 (2)	0.0640 (19)	0.068 (2)	0.0059 (16)	0.0216 (17)	0.0058 (15)
C33	0.078 (2)	0.0605 (19)	0.070 (2)	0.0130 (15)	0.0157 (17)	−0.0077 (16)
C34	0.0632 (18)	0.073 (2)	0.069 (2)	0.0064 (15)	0.0242 (15)	−0.0094 (16)
C35	0.0480 (15)	0.0676 (19)	0.0627 (17)	0.0014 (13)	0.0146 (13)	−0.0012 (14)
C36	0.208 (6)	0.084 (3)	0.144 (4)	0.060 (3)	0.071 (4)	0.033 (3)

### Geometric parameters (Å, º)

**Table:**

O1—C1	1.204 (4)	C24—C25	1.412 (5)
O2—C4	1.216 (4)	C24—C29	1.439 (4)
O3—C15	1.207 (4)	C25—C26	1.342 (5)
O4—C33	1.367 (4)	C26—C27	1.403 (6)
O4—C36	1.394 (6)	C27—C28	1.356 (5)
N1—C1	1.372 (3)	C28—C29	1.433 (5)
N1—C3	1.479 (3)	C30—C31	1.364 (4)
N1—C30	1.409 (3)	C30—C35	1.383 (4)
N2—C2	1.439 (4)	C31—C32	1.398 (4)
N2—C4	1.391 (4)	C32—C33	1.375 (4)
N2—C15	1.409 (4)	C33—C34	1.367 (5)
C1—C2	1.538 (4)	C34—C35	1.380 (4)
C2—C3	1.561 (4)	C2—H2	0.9800
C3—C16	1.511 (3)	C3—H3	0.9800
C4—C5	1.468 (4)	C6—H6	0.9300
C5—C6	1.364 (4)	C7—H7	0.9300
C5—C10	1.406 (4)	C8—H8	0.9300
C6—C7	1.424 (5)	C12—H12	0.9300
C7—C8	1.350 (6)	C13—H13	0.9300
C8—C9	1.402 (6)	C14—H14	0.9300
C9—C10	1.422 (5)	C18—H18	0.9300
C9—C14	1.407 (6)	C19—H19	0.9300
C10—C11	1.403 (4)	C20—H20	0.9300
C11—C12	1.387 (5)	C21—H21	0.9300
C11—C15	1.474 (4)	C23—H23	0.9300
C12—C13	1.390 (6)	C25—H25	0.9300
C13—C14	1.369 (7)	C26—H26	0.9300
C16—C17	1.401 (4)	C27—H27	0.9300
C16—C29	1.420 (4)	C28—H28	0.9300
C17—C18	1.429 (5)	C31—H31	0.9300
C17—C22	1.449 (4)	C32—H32	0.9300
C18—C19	1.353 (5)	C34—H34	0.9300
C19—C20	1.395 (6)	C35—H35	0.9300
C20—C21	1.339 (6)	C36—H36A	0.9600
C21—C22	1.419 (5)	C36—H36B	0.9600
C22—C23	1.378 (5)	C36—H36C	0.9600
C23—C24	1.381 (5)		
			
C33—O4—C36	118.3 (3)	C16—C29—C28	124.5 (3)
C1—N1—C3	95.5 (2)	C24—C29—C28	115.9 (3)
C1—N1—C30	132.1 (2)	N1—C30—C31	120.2 (2)
C3—N1—C30	129.9 (2)	N1—C30—C35	120.4 (2)
C2—N2—C4	118.0 (2)	C31—C30—C35	119.4 (3)
C2—N2—C15	117.0 (2)	C30—C31—C32	120.3 (3)
C4—N2—C15	125.0 (2)	C31—C32—C33	119.9 (3)
O1—C1—N1	132.5 (3)	O4—C33—C32	124.6 (3)
O1—C1—C2	136.2 (3)	O4—C33—C34	115.8 (3)
N1—C1—C2	91.3 (2)	C32—C33—C34	119.6 (3)
N2—C2—C1	120.1 (2)	C33—C34—C35	120.5 (3)
N2—C2—C3	119.1 (3)	C30—C35—C34	120.3 (3)
C1—C2—C3	85.88 (19)	N2—C2—H2	110.00
N1—C3—C2	86.47 (19)	C1—C2—H2	110.00
N1—C3—C16	121.6 (2)	C3—C2—H2	110.00
C2—C3—C16	117.1 (2)	N1—C3—H3	110.00
O2—C4—N2	119.0 (3)	C2—C3—H3	110.00
O2—C4—C5	124.0 (3)	C16—C3—H3	110.00
N2—C4—C5	117.1 (3)	C5—C6—H6	120.00
C4—C5—C6	120.0 (3)	C7—C6—H6	120.00
C4—C5—C10	120.2 (3)	C6—C7—H7	120.00
C6—C5—C10	119.8 (3)	C8—C7—H7	120.00
C5—C6—C7	119.8 (3)	C7—C8—H8	119.00
C6—C7—C8	120.7 (4)	C9—C8—H8	119.00
C7—C8—C9	121.2 (4)	C11—C12—H12	120.00
C8—C9—C10	118.1 (3)	C13—C12—H12	120.00
C8—C9—C14	122.9 (4)	C12—C13—H13	120.00
C10—C9—C14	119.0 (3)	C14—C13—H13	120.00
C5—C10—C9	120.3 (3)	C9—C14—H14	119.00
C5—C10—C11	120.9 (3)	C13—C14—H14	119.00
C9—C10—C11	118.7 (3)	C17—C18—H18	119.00
C10—C11—C12	120.5 (3)	C19—C18—H18	119.00
C10—C11—C15	120.4 (3)	C18—C19—H19	119.00
C12—C11—C15	119.1 (3)	C20—C19—H19	119.00
C11—C12—C13	120.4 (4)	C19—C20—H20	120.00
C12—C13—C14	120.1 (4)	C21—C20—H20	120.00
C9—C14—C13	121.1 (4)	C20—C21—H21	119.00
O3—C15—N2	120.2 (3)	C22—C21—H21	119.00
O3—C15—C11	123.4 (3)	C22—C23—H23	119.00
N2—C15—C11	116.4 (3)	C24—C23—H23	119.00
C3—C16—C17	117.6 (2)	C24—C25—H25	119.00
C3—C16—C29	122.2 (2)	C26—C25—H25	119.00
C17—C16—C29	119.8 (2)	C25—C26—H26	120.00
C16—C17—C18	124.8 (3)	C27—C26—H26	120.00
C16—C17—C22	119.5 (3)	C26—C27—H27	120.00
C18—C17—C22	115.7 (3)	C28—C27—H27	120.00
C17—C18—C19	121.8 (3)	C27—C28—H28	119.00
C18—C19—C20	121.7 (4)	C29—C28—H28	119.00
C19—C20—C21	119.6 (4)	C30—C31—H31	120.00
C20—C21—C22	121.7 (3)	C32—C31—H31	120.00
C17—C22—C21	119.5 (3)	C31—C32—H32	120.00
C17—C22—C23	119.6 (3)	C33—C32—H32	120.00
C21—C22—C23	120.9 (3)	C33—C34—H34	120.00
C22—C23—C24	122.0 (3)	C35—C34—H34	120.00
C23—C24—C25	120.6 (3)	C30—C35—H35	120.00
C23—C24—C29	119.4 (3)	C34—C35—H35	120.00
C25—C24—C29	120.0 (3)	O4—C36—H36A	110.00
C24—C25—C26	121.2 (3)	O4—C36—H36B	110.00
C25—C26—C27	120.1 (4)	O4—C36—H36C	109.00
C26—C27—C28	120.9 (3)	H36A—C36—H36B	109.00
C27—C28—C29	121.7 (3)	H36A—C36—H36C	109.00
C16—C29—C24	119.6 (3)	H36B—C36—H36C	109.00
			
C36—O4—C33—C32	−9.3 (6)	C10—C9—C14—C13	0.7 (6)
C36—O4—C33—C34	172.3 (4)	C9—C10—C11—C15	177.4 (3)
C3—N1—C1—C2	−7.7 (2)	C5—C10—C11—C15	−1.6 (5)
C30—N1—C1—C2	−170.2 (3)	C9—C10—C11—C12	−2.8 (5)
C1—N1—C30—C31	152.4 (3)	C5—C10—C11—C12	178.2 (3)
C3—N1—C30—C31	−4.7 (4)	C15—C11—C12—C13	−177.9 (4)
C1—N1—C30—C35	−26.2 (4)	C12—C11—C15—O3	0.8 (5)
C3—N1—C30—C35	176.7 (3)	C10—C11—C12—C13	2.3 (6)
C3—N1—C1—O1	175.5 (4)	C12—C11—C15—N2	−178.6 (3)
C30—N1—C1—O1	12.9 (6)	C10—C11—C15—O3	−179.4 (3)
C30—N1—C3—C2	170.7 (2)	C10—C11—C15—N2	1.2 (4)
C1—N1—C3—C16	127.4 (3)	C11—C12—C13—C14	−0.2 (7)
C30—N1—C3—C16	−69.4 (4)	C12—C13—C14—C9	−1.3 (7)
C1—N1—C3—C2	7.6 (2)	C29—C16—C17—C18	177.7 (3)
C15—N2—C2—C1	144.1 (3)	C3—C16—C17—C22	170.5 (2)
C2—N2—C15—O3	0.3 (4)	C3—C16—C17—C18	−9.8 (4)
C15—N2—C4—C5	−4.3 (4)	C29—C16—C17—C22	−2.0 (4)
C4—N2—C2—C3	65.2 (3)	C3—C16—C29—C24	−168.8 (2)
C4—N2—C2—C1	−38.0 (4)	C17—C16—C29—C28	−176.8 (3)
C15—N2—C2—C3	−112.9 (3)	C17—C16—C29—C24	3.4 (4)
C4—N2—C15—O3	−177.5 (3)	C3—C16—C29—C28	11.0 (4)
C2—N2—C4—O2	−2.0 (4)	C16—C17—C22—C21	−178.3 (3)
C2—N2—C15—C11	179.7 (3)	C16—C17—C22—C23	−0.7 (4)
C2—N2—C4—C5	177.9 (3)	C18—C17—C22—C21	1.9 (5)
C4—N2—C15—C11	1.9 (4)	C18—C17—C22—C23	179.6 (3)
C15—N2—C4—O2	175.9 (3)	C22—C17—C18—C19	−1.0 (6)
O1—C1—C2—N2	−54.7 (5)	C16—C17—C18—C19	179.3 (4)
O1—C1—C2—C3	−176.1 (4)	C17—C18—C19—C20	−1.1 (8)
N1—C1—C2—N2	128.6 (3)	C18—C19—C20—C21	2.2 (9)
N1—C1—C2—C3	7.3 (2)	C19—C20—C21—C22	−1.1 (8)
N2—C2—C3—C16	107.0 (3)	C20—C21—C22—C23	−178.5 (4)
C1—C2—C3—N1	−6.7 (2)	C20—C21—C22—C17	−0.9 (6)
N2—C2—C3—N1	−129.1 (3)	C21—C22—C23—C24	179.6 (3)
C1—C2—C3—C16	−130.7 (3)	C17—C22—C23—C24	2.0 (4)
N1—C3—C16—C17	144.6 (3)	C22—C23—C24—C25	−179.6 (3)
C2—C3—C16—C17	−111.9 (3)	C22—C23—C24—C29	−0.6 (4)
C2—C3—C16—C29	60.4 (3)	C23—C24—C29—C16	−2.1 (4)
N1—C3—C16—C29	−43.1 (4)	C23—C24—C29—C28	178.1 (3)
N2—C4—C5—C10	3.7 (4)	C29—C24—C25—C26	3.4 (6)
O2—C4—C5—C6	4.0 (5)	C25—C24—C29—C28	−2.9 (4)
N2—C4—C5—C6	−175.8 (3)	C25—C24—C29—C16	176.9 (3)
O2—C4—C5—C10	−176.5 (3)	C23—C24—C25—C26	−177.6 (4)
C4—C5—C10—C9	−179.9 (3)	C24—C25—C26—C27	−1.2 (7)
C6—C5—C10—C11	178.7 (3)	C25—C26—C27—C28	−1.4 (7)
C10—C5—C6—C7	0.5 (5)	C26—C27—C28—C29	1.8 (6)
C4—C5—C6—C7	−180.0 (3)	C27—C28—C29—C24	0.4 (5)
C4—C5—C10—C11	−0.9 (5)	C27—C28—C29—C16	−179.4 (3)
C6—C5—C10—C9	−0.4 (5)	N1—C30—C35—C34	177.6 (3)
C5—C6—C7—C8	−0.7 (6)	C31—C30—C35—C34	−1.0 (4)
C6—C7—C8—C9	0.7 (7)	N1—C30—C31—C32	−178.2 (3)
C7—C8—C9—C10	−0.5 (6)	C35—C30—C31—C32	0.5 (4)
C7—C8—C9—C14	179.5 (4)	C30—C31—C32—C33	1.2 (5)
C8—C9—C10—C5	0.4 (5)	C31—C32—C33—C34	−2.3 (5)
C14—C9—C10—C5	−179.7 (3)	C31—C32—C33—O4	179.3 (3)
C14—C9—C10—C11	1.3 (5)	O4—C33—C34—C35	−179.7 (3)
C8—C9—C10—C11	−178.7 (3)	C32—C33—C34—C35	1.8 (5)
C8—C9—C14—C13	−179.3 (4)	C33—C34—C35—C30	−0.1 (5)

### Hydrogen-bond geometry (Å, º)

Cg5 is the centroid of the C16/C17/C22–C24/C29 benzene ring.

**Table:**

*D*—H···*A*	*D*—H	H···*A*	*D*···*A*	*D*—H···*A*
C28—H28···N1	0.93	2.35	3.022 (4)	129
C13—H13···*Cg*5^i^	0.93	2.84	3.713 (4)	158

Symmetry code: (i) −*x*, −*y*+1, −*z*+1.
