# Supplementary figures and images for: Crystal structure of 2-[(3S,4S)-4-(anthracen-9-yl)-1-(4-meth­oxy­phen­yl)-2-oxoazetidin-3-yl]-2-aza-2H-phenalene-1,3-dione unknown solvate
Source: Acta Crystallogr E Crystallogr Commun. 2015 Feb 13;71(Pt 3):o184–5. doi: 10.1107/S2056989015002959 (PMC4350708; doi:10.1107/S2056989015002959)

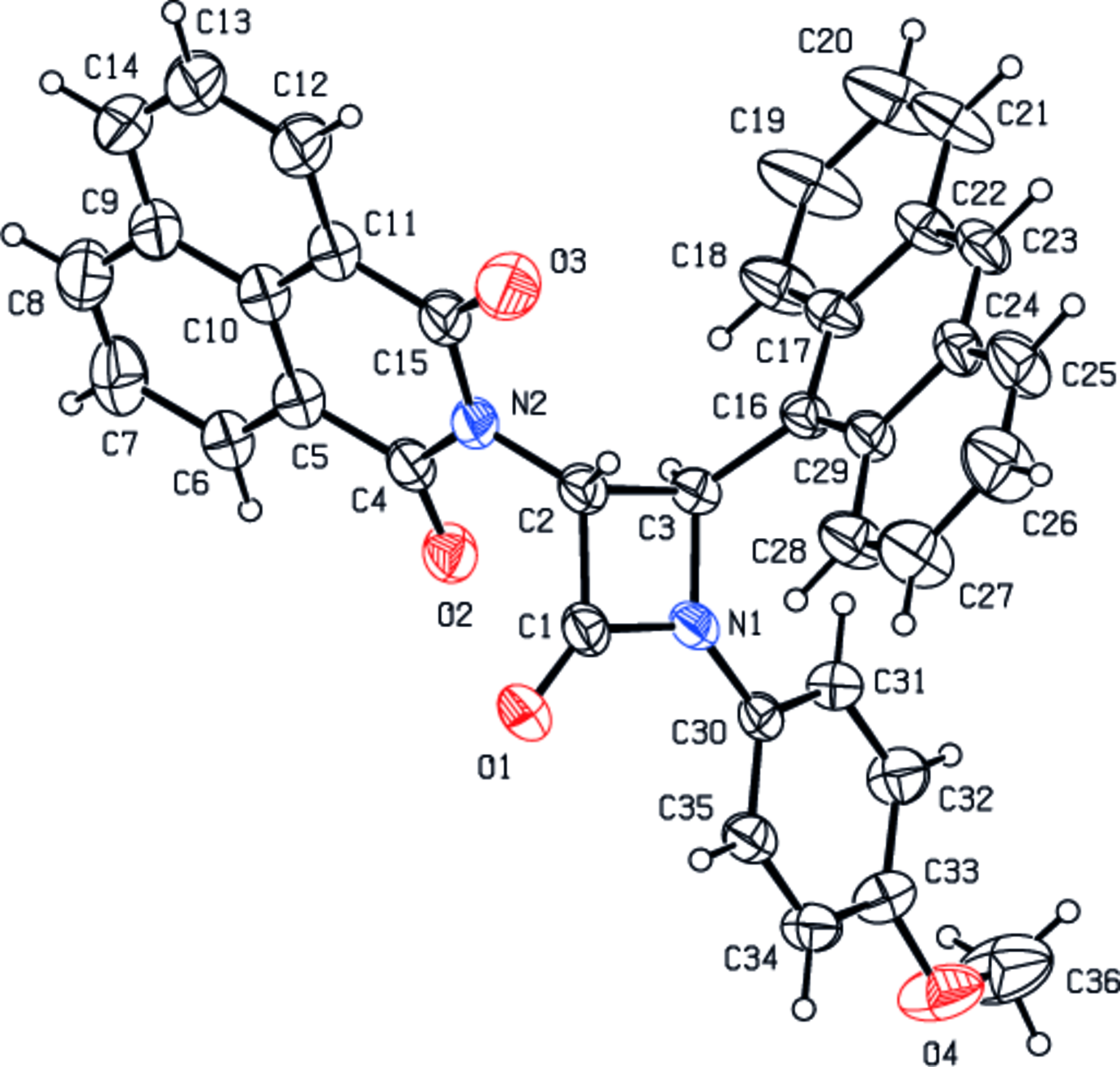

Supplement: Supplementary file 4 [file e-71-0o184-fig1.tif]

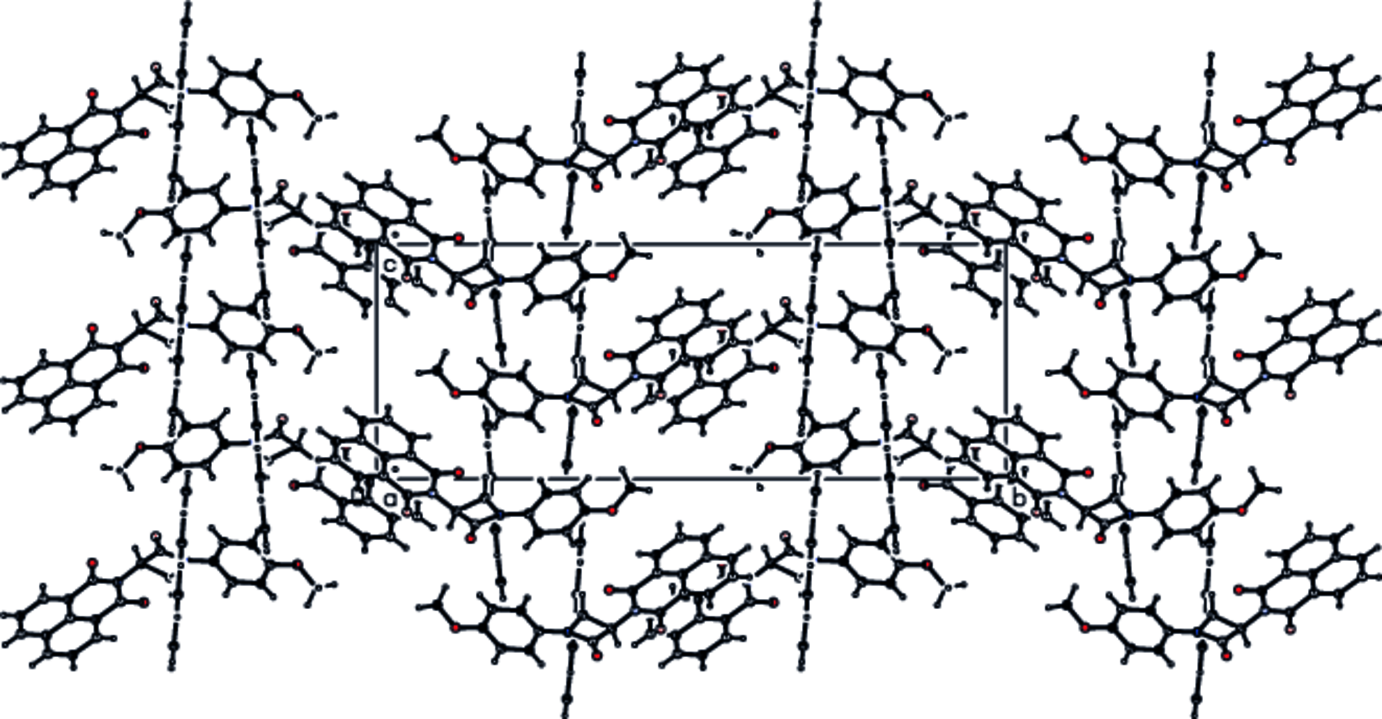

Supplement: Supplementary file 5 [file e-71-0o184-fig2.tif]
